# Quorum-driven microbial consortium for Bioplastic production from agro-waste

**DOI:** 10.1021/acssuschemeng.5c05453

**Published:** 2025-08-28

**Authors:** Diego Crespo-Roche, Marta Herráez, Javier Guerrero-Flores, M. Jesús Martínez, Katherine Louie, Trent Northen, Alicia Prieto, Jorge Barriuso

**Affiliations:** 1 Centro de Investigaciones Biológicas Margarita Salas, Consejo Superior de Investigaciones Científicas (CIB-CSIC) C/Ramiro de Maeztu 9, Madrid 28040, Spain; 2 Joint Genome Institute, 1666 Lawrence Berkeley National Laboratory, Berkeley, California 94720, United States

**Keywords:** Polyhydroxyalkanoates, breweries spent grain, waste cooking oil, Ophiostoma, Pseudomonas, quorum sensing, farnesol

## Abstract

Microbial consortia have high relevance in natural environments. Here we present the production of polyhydroxyalkanoates (PHA) from agro-industrial residues by a synthetic interkingdom consortium formed by the saprotrophic fungus *Ophiostoma piceae* CECT 20146, which encodes a wide range of lignocellulolytic enzymes, and a natural PHA producer, *Pseudomonas putida* KT2440. Two agro-industrial residues were utilized: Brewer’s Spent Grain (BSG) as a carbon/nitrogen source and biofilm scaffold and waste cooking oil (WCO) as a carbon source for PHA synthesis. Through biochemistry, microscopy, and omics analyses, it is shown that *P. putida* accumulates up to 40.2% of intracellular PHA when the quorum sensing molecule, farnesol (naturally produced by *O. piceae*) is added, thanks to the increased proliferation of *P. putida* cells. An interactive Shiny application has also been developed for an easy visualization and comprehension of all the transcriptomics and metabolomics data: https://jgf-bioinformatics.shinyapps.io/Visualization_app/. These results support the increased PHA production of the consortium by an induction of gene *phaG*, which redirects intermediaries of the fatty acid biosynthesis to PHA precursors, and the repression of the PHA depolymerase *phaZ* in *P. putida*. The trophic interaction between microorganisms seems to rely on the citric acid produced by *O. piceae* and the glycerol liberated from WCO, which can both be consumed by *P. putida*. Bioreactor scale-up experiments allowed a 3.3-fold increase in the PHA concentration in the consortium (6.7 g·L^–1^) without pretreatment or sterilization of the substrates, laying the groundwork for the implementation of an industrial consolidated bioprocess (CBP).

## Graphical abstract

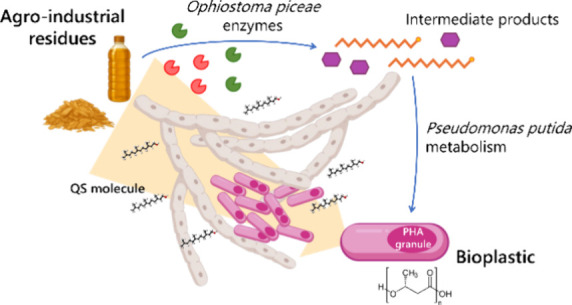

## Introduction

Conventional plastics, derived from fossil fuels, have high versatility to meet a wide range of needs in modern life. However, the processes involved in their production are highly polluting, and most of them are hardly biodegradable and remain in the environment for decades or centuries.^1^ In this context, bioplastics have emerged as a sustainable alternative, as they are polymeric materials that are either derived from renewable sources, are biodegradable, or possess both properties.^2^ Among them, polyhydroxyalkanoates (PHAs), polyesters of 3-, 4-, 5- and 6-hydroxyalkanoic acids, stand out for fulfilling both defining criteria of a Bioplastic. These polymers are also biocompatible, and depending on their composition, have similar properties to conventional thermoplastics and elastomers.^3^ PHAs are synthesized as energy and carbon storage material by a variety of bacteria and their accumulation occurs under unfavorable growth conditions such as nutritional stress.^4^ The major drawback preventing wide commercial use of these polymers resides in their high production costs, as the substrates used for industrial production, such as glucose, are expensive. The development of competitive and efficient strategies for PHA production from cheaper substrates will allow its entry into the market.^3^

In this context, agro-industrial residues, which are massively produced by agriculture-based industries and that are commonly untreated and underutilized, have a great potential as secondary raw materials.^5,6^ Brewers’ spent grain (BSG) is the major residue produced by the brewing industry (20 kg for every 100 L of beer produced).^7^ Its low cost, large availability throughout the year, valuable chemical composition (cellulose, hemicellulose, lignin, protein, and lipids) and physical characteristics such as particle size, specific density and porosity, make BSG a valuable resource for microbial bioconversion into value-added products.^8,9^ However, the use of lignocellulosic biomass requires a physicochemical pretreatment to remove the lignin barrier and allow enzymes to act on polysaccharides to release sugar monomers.^10^ BSG hydrolysates have been used to produce xylitol by the yeast *Candida guilliermondii*,^11^ intracellular fatty acids and carotenoids by the yeast *Rhodosporidium toruloides*,^12^ and lactic acid by several species of *Lactobacillus*.^13,14^ On the other hand, waste cooking oil (WCO, oils produced in the food cooking process), composed mainly of triglycerides, is a feedstock with high industrial relevance.^15^ Moreover, its release to urban water recycling systems can produce severe environmental pollution.^16^

In nature, microbes are mainly responsible for the transformation of lignocellulosic substrates. Saprophytic fungi and some bacteria (mainly Proteobacteria) serve as the primary degraders of lignocellulose through the action of extracellular enzymes such as peroxidases, laccases, lipases, cellulases and hemicellulases.^17–19^ To date, most industrial fermentations are carried out by microbial monocultures, without exploiting the potential of microbial communities, their associations and the division of labor that takes place within them.^20–22^ Recently, mixed consortia comprising eukaryotic and prokaryotic organisms have been explored to produce compounds of industrial importance. Some examples are 2-keto-L-gulonic acid, synthesized in cocultures of a genetically engineered strain of *Saccharomyces cerevisiae* and *Ketogulonigenium vulgare*;^23^ naringenin, produced by a coculture of an engineered strain of *S. cerevisiae* and *E. coli*;^24^ butyric acid, produced from lignocellulosic biomass by two synthetic consortia formed by *Trichoderma reesei* and lactic acid bacteria;^25^ or lactic acid, released directly from starch residues in cocultures of *Talaromyces amestolkiae* and *Lactiplantibacillus plantarum*.^26^

In nature, communication between species in a microbial consortium is key to success. Quorum sensing (QS) is a sophisticated, density-dependent cell-to-cell communication system mediated by small molecules, denominated quorum sensing molecules (QSMs) or autoinducers, that coordinate the behavior of the population. QS communication occurs not only among individuals of the same species, but also from different species and kingdoms.^27^

The ascomycete fungus *Ophiostoma piceae* CECT 20416 is a wood saprotroph whose genome encodes 382 putative enzymes active on carbohydrates and lignin, allowing the degradation of lignocellulosic materials such as BSG.^28^ Moreover, it secretes the versatile lipase OPE,^29^ very active on triglycerides and sterol esters.^30^ QS mechanism described in *O. piceae* is mediated by the molecule *E,E*-farnesol, which regulates the morphological transition from yeast to hyphae,^28^ biofilm formation, and extracellular esterase activity.^31^

On the other hand, the bacterium *Pseudomonas putida* KT2440 is characterized by its broad metabolic versatility,^32,33^ what makes it a flexible chassis for metabolic engineering and production of biobased chemicals as PHAs.^32^ *P. putida* does not encode extracellular lignocellulolytic enzymes, so its growth in BSG is impaired.^34^ However, this bacterium can metabolize degradation products (monosaccharides, organic acids, phenols) released from plant biomass.^35^ QS mechanisms have been described in *P. putida*, presenting an orphan Lux-type QS system mediated by homoserine lactones,^36^ and a RoxS/RoxR QS system mediated by dodecanoic acid.^37^ In addition, this bacterium responds to farnesol (the QS molecule secreted by *O. piceae*), stimulating the formation of biofilms. In this sense, the formation of mixed *O. piceae* – *P. putida* biofilms modulated by QS mechanisms has been reported.^38^

In this work, we address the formation of a synthetic interkingdom consortium for the one-step bioconversion of a mixture of two residues (BSG and WCO) into PHAs. This approach aims to replace multistep industrial processes, reducing pretreatments and fermentations costs, improving efficiency, and increasing competitiveness. The experimental design involves a consolidated bioprocess (CBP) mediated by a coculture of the dimorphic fungus *O. piceae,* able to degrade the selected wastes, and *P. putida,* able of producing PHA from the released substrates. The effect of the QS molecule farnesol on the formation of fungal-bacterial catalytic biofilms and on PHA production is also assessed. The association of *O. piceae* and *P. putida* is investigated using biochemical, microscopy, and omics techniques, obtaining extensive and multifaceted information on the relationship established between these two microorganisms in terms of physical interactions, trophic dynamics, and cell-to-cell communication.

## Materials and methods

### Chemicals, reagents and wastes

Brewers’ spent grain (BSG) was sourced from ″La Cibeles″ brewery in Madrid and conserved at −20 °C until required. For BSG pretreatment, the frozen sample was thawed, dried at 45 °C, and subsequently milled and sieved to a particle size <420 μm. The BSG underwent a mild alkaline pretreatment in 0.5% (w/v) NaOH for 1 h at 50 °C, with a solid-to-liquid ratio of 1:20. The resultant mixture was neutralized using 72% H_2_SO_4_ and freeze-dried for utilization in the flask experiments. Domestic cooking olive oil waste was autoclaved upon receipt and stored until it was needed. The QS molecule *trans,trans*-farnesol (FaOH) was purchased from Sigma-Aldrich (ref 277541).

### Microorganisms, culture media, and growth conditions

The microorganisms used in this study were *O. piceae* CECT 20146 (obtained from the fungal collection available in the laboratory at Center for Biological Research, CIB-CSIC) and *P. putida* KT2440 (kindly provided by Dr. Víctor de Lorenzo, National Center for Biotechnology, CNB-CSIC). Fungal preinocula were prepared cultivating *O. piceae* for 72 h at 180 rpm and 28 °C in glucose phosphate proline (GPP) minimal medium, containing 20 g·L^–1^ of glucose.^28^ Before inoculation, the cultures were filtered through sterile Miracloth (EMD Millipore) to remove hyphae. The resulting yeast suspension was centrifuged (10 min, 13000 *g*), washed, and resuspended in phosphate buffered saline (PBS, pH 6.8) to determine cell concentration using a Thoma counting chamber under the microscope. In the case of *P. putida,* the bacterium was cultivated overnight in Lysogeny broth (LB) medium at 180 rpm and 30 °C, the preinoculum was centrifuged to recover the pellet, that was washed and resuspended in PBS to determine cell concentration using a Thoma counting chamber.

For PHA production, the culture medium used was a modified GPP in which glucose was replaced with a mixture containing 1% pretreated BSG (w/v) and 1% WCO (v/v) as carbon sources. The microorganisms were cultivated as monocultures and in consortium in 250 mL Erlenmeyer flasks with 50 mL of the medium, using noninoculated flasks as controls. *O. piceae* was inoculated to a final concentration of 10^6^ cells·mL^–1^, while *P. putida* was inoculated to a final concentration of 10^5^ cells·mL^–1^ 24 h after fungal inoculation.

To assess the effect of farnesol, this QS molecule was added at a final concentration of 75 μM also 24 h after fungal inoculation. The inoculation conditions and final farnesol concentration are based in previous findings made in Ruiz et al.^38^ Each condition, with or without farnesol, was subjected to triplicate testing. Experiments were performed for 192 h at 28 °C and 180 rpm.

### Determination of microbial growth

Every 24 h, a 1 mL aliquot was extracted from each Erlenmeyer flask using 5 mL sterile plastic pipet tips. The end of the tips was cut out to ensure the collection of both BSG particles and mycelium when the fungus is present. To quantify culture growth, optical density at 600 nm was assessed by using a Shimadzu UV-1900i UV–vis spectrophotometer. Then, samples were filtered through a preweighed Miracloth filter, which was dried at 55 °C for 24 h to quantify the retained biomass dry weight. The particles retained by the filter comprisedBSG particles, mycelium, and/or biofilm components larger than 25 μm, depending on the culture condition. Subsequently, the OD of the flowthrough, containing bacteria and/or yeast, as confirmed by optical microscopy, was also determined.

### Determination of yeast and hyphae populations

Before filtration, samples from the flasks inoculated with *O. piceae* were observed in an optical Axioskop 2 Plus (Carl Zeiss GmBH) microscope with a 40x objective. Yeast and hyphae were counted independently in four different areas for each replicate. Cells with buds were counted as yeasts, and spores and cells forming germ tubes were classified as hyphae, as previously reported.^39^

### Flow cytometry

To determine the proportions of yeast and bacterial planktonic cell populations, the cultures filtered through Miracloth were permeabilized with ethanol 70% for 30 min at 4 °C and incubated with propidium iodide (1 μg·mL^–1^) to stain DNA. The particles were analyzed in the Flow Cytometry Facility at the Centre of Biological Research (CIB-CSIC, Madrid, Spain) in a Cytoflex-S (Beckman-Coulter) flow cytometer with excitation at 561 nm and fluorescence detection at 610/10 nm. Fluorescence, forward scatter area (FSC-A) and side scattering area (SSC-A) data were collected from a total of 50,000 events per sample.

### Confocal and Electron Microscopy

The structure of the microbial consortia formed on the BSG particles was studied by confocal laser scanning microscopy (CLSM), scanning electronic microscopy (SEM) and transmission electron microscopy (TEM).

CLSM was performed with a Leica TCS SP8 STED instrument in the Laser Confocal and Multidimensional *in vivo* Microscopy facility (LCMF) at the Centre of Biological Research (CIB-CSIC, Madrid, Spain). Samples were stained with 12.5 μg·mL^–1^ of Calcofluor White (CFW), 0.25 μg·mL^–1^ of BODIPY 493/503 (Sigma-Aldrich) and 5 μg·mL^–1^ of propidium iodide, after permeabilization at 4 °C with ethanol 70% for 30 min). Observations were conducted using a 100x objective with excitation wavelengths of: 405 nm for CFW (fluorescence collected within the range of 412–458 nm), 488 nm for Bodipy (fluorescence collected within the range of 508–546 nm), and 536 nm for propidium iodide (fluorescence collected within the range of 565–686 nm). The images were processed with Leica Application Suite X (LasX software).

SEM was performed by using a JEOL 6400 JSM scanning electron microscope with an acceleration voltage of 20 kV at the National Centre for Electronic Microscopy (CNME, Madrid, Spain). Samples of 75 μL were fixed using 425 μL of 2.5% glutaraldehyde for 4 h, following a drying procedure involving incremental concentrations of ethanol (30%, 50%, 80%, 90%, and 100%). The samples were then filtered through polytetrafluoroethylene filters (Fluoropore PTFE, EMD Millipore, FGLP01300). The filtered samples underwent a critical point process and gold metallization for visualization.

To observe the intracellular accumulation of PHAs at the final sampling time, samples were analyzed by TEM in the Electron Microscopy Facility (EMF) at the Centre of Biological Research (CIB-CSIC, Madrid, Spain). Cultures were fixated with a solution of 4% (w/v) paraformaldehyde and 2.5% (w/v) glutaraldehyde in phosphate buffer 50 mM pH 6.8 for 4 h at 4 °C. Then, the cells were rinsed twice with phosphate buffer (10–15 min), resuspended in 1% (w/v) OsO_4_ for 1 h, and washed 4 times for 10 min with Milli-Q water. Subsequently, the sample was dehydrated with increasing concentrations of acetone (30%, 50%, 80%, 90%, 95% and 100% (v/v)) for 10–15 min each, embedded in LX112 resin, and maintained at 70 °C for 48 h. Finally, using a microtome with a Diatome diamond knife, sections with of 70 nm-thickness were cut and deposited on carbon-coated copper grids to be finally observed in a JEOL JEM-1230 electron microscope.

### Esterase activity

The enzyme crude extracts were prepared by centrifugation and filtration of the culture supernatants. The extracellular esterase activity of the cultures was determined by a colorimetric assay following the release of *p*-nitrophenol from *p*-nitrophenyl butyrate (*p*NPB) at 410 nm for 3 min. The assay was performed at room temperature using 1.5 mM *p*NPB in 25 mM Tris-HCl as the substrate, to which 20 μL of crude extract was added (diluted when required) and measured in a Shimazdu UV-1900i UV–vis spectrophotometer. Activity is expressed in mU·mL^–1^, which refers to the amount of enzyme that releases 1 μmol of *p*-nitrophenol in one min per mL of reaction.

### Determination of PHA content

The determination of PHA content and composition in the cultures and filtered cells was conducted by GC-MS after methanolysis of the samples. In brief, lyophilized pellets of the cultures (10 mg of dry biomass) were incubated (100 °C for 5 h) with a mixture of 2 mL of 15% sulfuric acid in methanol and 2 mL chloroform containing 0.5 mg·mL^–1^ 3-methyl benzoate (the internal standard). Then, the sample was washed twice with Milli-Q H_2_O. The organic phase, containing the methyl esters and the internal standard was analyzed by GC-MS as described in de Eugenio et al.^40^ The final PHA concentration in the cultures was calculated from a calibration curve of PHA processed and analyzed in the same conditions as the samples.

### Transcriptomic analysis

The different conditions analyzed in this study for PHA production (*O. piceae* monocultures, *P. putida* monocultures, and the consortium; all three induced or not with farnesol) were subjected to transcriptomic studies at two sampling-points. RNA extraction was performed at 96 and 168 h of culture with RNeasy Plant Mini Kit (Qiagen) followed by a DNase treatment with TURBO DNA-free (Thermo Scientific). Libraries preparation and sequencing were carried out at the facilities of the Joint Genome Institute (JGI-DOE, USA). rRNA was depleted from 100 ng of total RNA using QIAseq FastSelect 5*S*/16*S*/23S and rRNA Yeast Kits (QIAgen). Using TruSeq stranded mRNA kit (Illumina), the 300 bp -400 bp heat fragmented RNA was reverse transcribed to create the first strand of cDNA with random hexamers and SuperScript II Reverse Transcriptase (ThermoFisher Scientific) followed by second strand synthesis. The double stranded cDNA fragments were treated with A-tailing, ligation with JGI’s dual indexed adapters (IDT) and enriched using 10 cycles of PCR. The prepared libraries were quantified using KAPA Biosystems’ next-generation sequencing library qPCR kit and run on a Roche LightCycler 480 real-time PCR instrument. Sequencing of the flowcell was performed on the Illumina NovaSeq sequencer using NovaSeq XP V1.5 reagent kits, S4 flowcell, following a 2 × 151 indexed run recipe. The reads were aligned to each reference genome (*Pseudomonas putida* KT 2440 ^33^ and *Ophiostoma piceae* CECT 20416 ^28^) using HISAT2 version 2.1.0.^41^ FeatureCounts was used to generate the raw gene counts file.^42^ Lastly, DESeq2 (Bioconductor package) v.1.30.1 ^43^ was employed to normalize such counts, as well as calculate the fold changes. All data generated can be found in https://genome.jgi.doe.gov/portal/Ophpiccriptomics_4_FD/Ophpiccriptomics_4_FD.info.html.

Gene enrichment analyses were conducted using the topGo package in R. To comprehensively annotate and interpret the functional roles of genes within our data set, a custom Gene Ontology (GO) database derived from the genome annotations for *O. piceae* ^28^ and *P. putida* ^33^ was created. To ensure the robustness and reliability of our findings only data with a < 0.05 [pval] before applying an FDR adjustment were considered for further analysis.

### Metabolomic analysis

Control and farnesol-induced cultures: without inoculation, *P. putida* monoculture, *O. piceae* monoculture and consortium, were analyzed for metabolites accumulation in the supernatant after 1 week of growth. To do that, supernatants were extracted with two different solvents, methanol for polar and nonpolar metabolites, and a chloroform-based extraction for lipid analysis. Liquid chromatography was performed using an Agilent 1290 LC stack, with MS and MS/MS data collected using a Thermo QExactive Orbitrap MS instrument (Thermo Scientific, Waltham, MA). Full MS spectra were collected at 70,000 resolution in both positive and negative ion modes, with MS/MS fragmentation data acquired using stepped 10, 20, and 40 eV collision energies at 17,500 resolution. Mass range varied depending on chromatography implemented, with *m*/*z* 70–1050 for polar HILICZ, 80–1200 for nonpolar C18, and 132–1500 for lipid C18. Mass spectrometer source settings included a sheath gas flow rate of 55 au (arbitrary units), auxiliary gas flow of 20 au, spray voltage of 3 kV (for both positive and negative ionization modes), and capillary temperature of 400 °C.

Chromatographic separation was performed using a HILICZ column (Agilent InfinityLab Poroshell 120, 2.1 × 150 mm, 2.7 μm, #673775–924) for polar metabolites and a C18 column (Agilent ZORBAX Eclipse Plus C18, Rapid Resolution HD, 2.1 × 50 mm, 1.8 μm, 95 Å, #959757–902) for nonpolar and lipid metabolites.

For polar metabolites, runs were carried out at 40 °C with 100% buffer B (99.8% 95:5 v/v ACN:H_2_O and 0.2% acetic acid, w/5 mM ammonium acetate) for 1 min, diluting buffer B down to 89% with buffer A (99.8% H_2_O, 0.2% acetic acid, 5 mM ammonium acetate, and 5 μM methylenediphosphonic acid) over 10 min, down to 70% B over 4.75 min, then down to 20% B over 0.5 min, followed by isocratic elution in 80% buffer A for 2.25 min at a flow rate of 0.45 mL/min with a 2-μL injection volume.

For nonpolar metabolites, runs were carried out at 60 °C with 100% buffer A (99.9% water w/0.1% formic acid) for 1 min, diluting buffer A down to 0% with buffer B (99.9% acetonitrile w/0.1% formic acid) over 7 min, followed by isocratic elution in 100% buffer B for 1.5 min at a flow rate of 0.4 mL/min with a 2-μL injection volume.

For lipid metabolites, runs were carried out at 55 °C with 100% buffer A (99.9% 60:40 water:acetonitrile and 0.1% formic acid with 5 mM ammonium acetate) for 1 min, diluting buffer A down to 40% with buffer B (″99.8% 90:10 isopropanol:acetonitrile, 0.1% water, and 0.1% formic acid with 5 mM ammonium acetate″) over 2 min, further diluting A down to 20% over 8 min, then down to 0% over 5 min, followed by isocratic elution in 100% buffer B for 1.5 min at a flow rate of 0.4 mL/min with a 2-μL injection volume.

For metabolites identification, a Feature-Based Molecular Networking workflow was used (version release 18),^44^ employing MZmine2 ^45^ and Global Natural Products Social Molecular Networking (GNPS)^46^ for putative identifications by matching sample spectra with entries in the GNPS database. Results were filtered by a cosine similarity of 0.7 and a peak height of 1 × 10^6^. The GNPS also provides with a chemical taxonomy classification of the features identified that was used for the metabolite’s classification in classes. Metabolomics raw data are deposited in the MassIVE data repository (https://massive.ucsd.edu/).

### Omics data processing

A web application was developed using Shiny^47^ in R to facilitate transcriptomics and metabolomics data exploration and visualization due to the large volume of information generated. The code and user instructions can be found at: https://github.com/JavierGuerreroF/Transcriptomics-metabolomics-visualization-app-KTOphi?tab=readme-ov-file. The application allows users to interactively explore the data collected from our experiments (https://jgf-bioinformatics.shinyapps.io/Visualization_app/). Within the transcriptomics tab, users can browse the gene expression patterns and make comparisons across different experimental conditions. Results are shown as volcano plots that allow users to identify over and underexpressed genes using customizable filters such as log fold change and p-value. Moreover, the gene-specific search function enables users to retrieve and display fold changes for a particular gene across the various conditions.

Metabolomic data are represented as the proportional distribution of metabolites, classified based on the Feature-Based Molecular Networking analysis^44^ available on the GNPS^46^ Web site. Users can explore the different classes of metabolites and search for specific metabolites in each sample. Additionally, the app allows the comparison of different conditions and filtering of metabolites by peak height and choosing alternative classifications for a more refined analysis.

### Bioreactor scaling-up

The best performing shake-flask culture conditions (consortium induced with farnesol) were replicated in a 5 L bioreactor (Aplikon BioBundle3M) with a 2-L jacketed vessel, a dual Rushton 6-bladed impeller arrangement, and an optical sensor integrated with an Ez-2control station, with meticulous control over temperature (28 °C), pH (5.8), stirring (400 rpm), and aeration (2 L·L^–1^·min^–1^) parameters. BSG and WCO were added at 2% (w/v) and 2% (v/v), respectively. BSG was not pretreated or sterilized with the aim of optimizing the consolidated bioprocess. The inoculation was performed with 10^7^ cells·mL^–1^ for both microorganisms without any time delay, farnesol was added 24 h postinoculation. The growth of the cocultures and PHA production were monitored as mentioned above.

## Results and discussion

### Microbial growth and morphology

Growth was determined by measuring OD_600 nm_ in the cultures before and after filtration through Miracloth and by measuring the dry weight of the biomass retained in the filter (Figure 1). The filtrate contained individual cells (bacteria and yeast) and the retentate hyphae and BSG particles. The OD_600 nm_ in *P. putida* monocultures was very similar in both filtered and unfiltered cultures confirming that bacteria were not retained, reaching an OD_600 nm_ of 4 at 24 h of bacterial growth (48 h from the beginning of the experiment) and remaining stable until the end of the experiment. In addition, the bacterial biomass retained in the filter did not vary (1–2 mg·mL^–1^) during the whole experiment (Figure 1C), suggesting that the bacterium grows moderately in this substrate, as it cannot degrade BSG particles and can only metabolize soluble metabolites, independently of the addition of farnesol.

**Figure 1:**
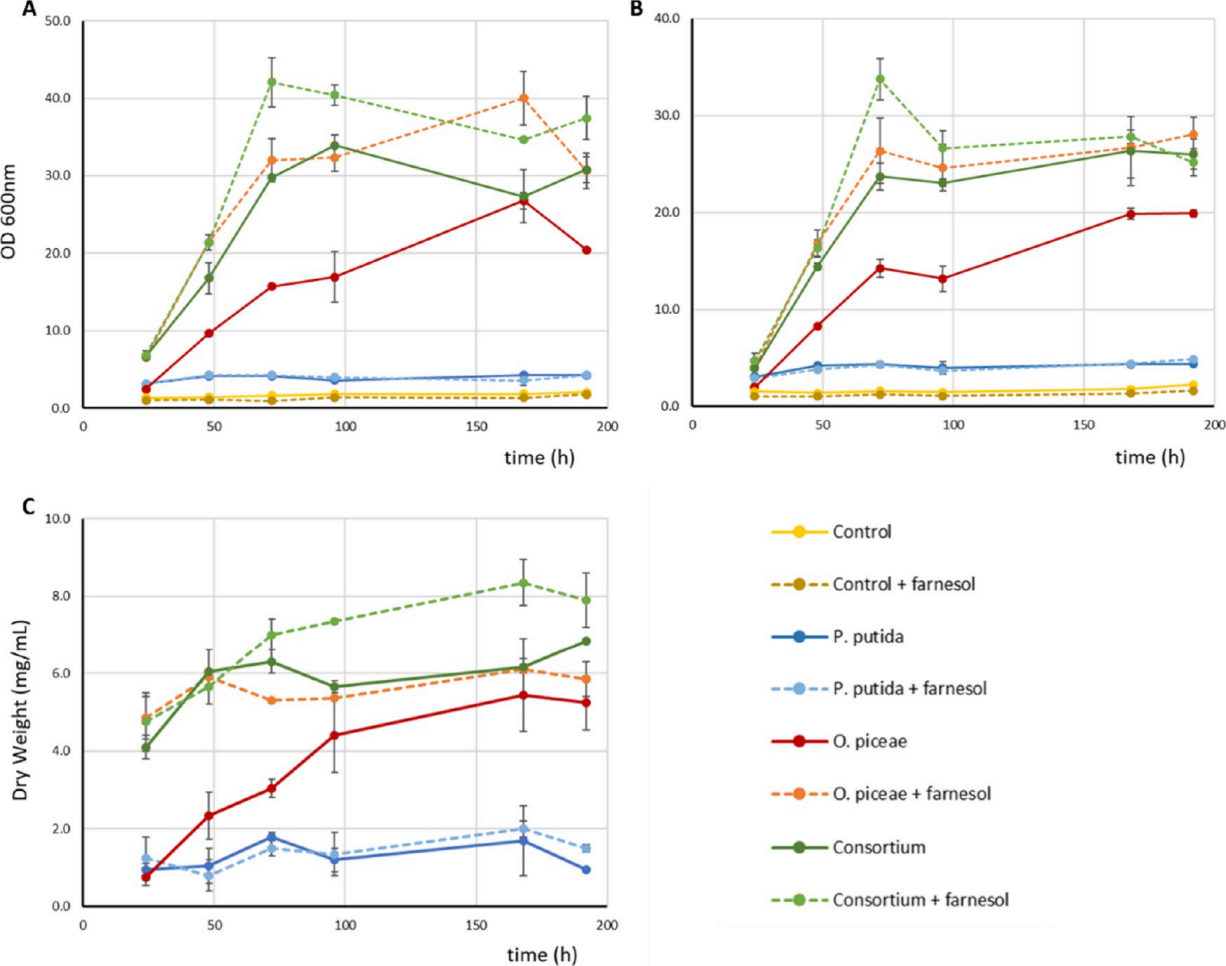
Evolution of growth (OD_600 nm_) in pure cultures of *P. putida* and *O. piceae* and their coculture: A) before filtration through Miracloth and B) after filtration. C) Dry weight of the biomass recovered from the Miracloth filter. Legend: noninoculated control (yellow line), noninoculated control with farnesol (dark yellow dotted line), *P. putida* monoculture (dark blue line), *P. putida* monoculture with farnesol (blue dotted line), *O. piceae* monoculture (red line), *O. piceae* monoculture with farnesol (orange dotted line), consortium (dark green) and consortium with farnesol (green dotted line). Error bars represent standard error for 3 biological replicates.

In contrast, the growth in *O. piceae* monocultures and in the *P. putida-O. piceae* consortium was high and increased with the addition of farnesol. The growth profiles of fungal monocultures with and without the QSM were similar and reached the stationary phase at 168 h, but their maximum OD_600 nm_ and the dry weight of the biomass retained in the filter were very different. In the case of the consortium, farnesol induced higher total OD_600 nm_ and earlier stationary phase of the cultures (OD 42 and 72 h vs OD 34 and 96 h) (Figure 1A). In addition, the larger amount of biomass recovered from the filters in induced fungal monocultures and consortia can be related to an increase in the hypha proportion during the growth phase and biofilm formation around the BSG particles. The greatest differences in the proportion of hyphae and yeast cells investigated under the light microscope were detected at 72 h of growth, when fungal monocultures contained 9.6 ± 2.2% hyphae, and the value increased to 25.5 ± 3.8 in cultures induced with farnesol. In the consortium, these proportions amounted to 27.8 ± 2.6% and 31.0 ± 2.8%, without and with farnesol, respectively. The maximum level of filamentation among all samples was attained in the induced consortium (69.1 ± 11.6%) at 96 h, confirming in all cases the positive impact of farnesol on filamentation.

Furthermore, the morphology and structure of the biofilms formed in the BSG particles were observed by SEM and CLSM. *P. putida* showed limited colonisation of the BSG particles and improved with farnesol addition (Figure 2A-B), which agrees with the low amount of biomass recovered from the Miracloth filter in these cultures (Figure 1C). On the contrary, biofilm was observed on BSG particles in *O. piceae* monocultures, with a higher colonization in the presence of farnesol (Figure 2D) due to an in increased hyphal proportion, as previously described.^27^ SEM images of the consortium revealed the formation of mixed biofilms with higher proportion of hyphae in the farnesol induced cultures; bacteria were attaching to fungal yeast, hyphae, and BSG particles (Figure 2E-F). These observations agree with our previous findings on the induction of filamentation and biofilm formation in *O. piceae* by the QSM farnesol^31^ and with filamentation measurements in this study.

**Figure 2:**
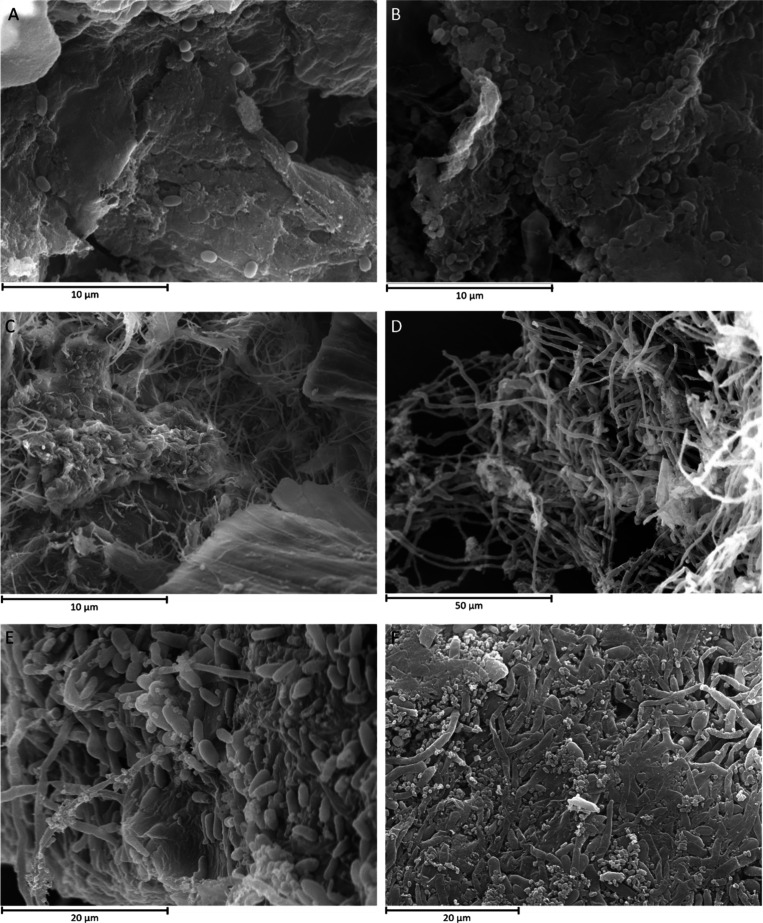
Scanning electron microscopy (SEM) images of cultures with BSG, with and without farnesol. A) *P. putida* (x5000), B) *P. putida* induced with farnesol (x5000), C) *O. piceae* (x5000), D) *O. piceae* induced with farnesol (x1000), E) *P. putida-O. piceae* consortia (x2500), and F) *P. putida-O. piceae* consortia induced with farnesol (x2000).

For better visualization of the organization of the main components of the farnesol-induced mixed biofilms, there were analyzed by CLSM (Figure 3A). Hyphae and yeast cell walls could be observed by staining with calcofluor, while bacterial and yeast DNA was stained with propidium iodide. Notably, hyphae did not stain with propidium iodide, probably due to the different composition of the cell envelope (ergosterol, chitin, β-1–3 and β-1–6 glucans), which may affect permeability.^28^ CLSM observations suggested a strong fungal-bacterial association, with a high proportion of hyphae, some yeasts, and bacteria in planktonic form, while other bacteria were attaching to the mycelium and forming aggregates (Figure 3A). *P. putida* cells also presented green intracellular fluorescence due to the presence of granules stained with BODIPY, which presumably correspond to PHA accumulation. *O. piceae* cells also showed big green drops, especially in hyphae, which can correspond to lipid droplets that some filamentous fungi produce as storage substances.^48^ *P. putida* cells filled with PHA granules were also observed by TEM (Figure 3B), and transversely cut yeasts and hyphae were also observed.

**Figure 3:**
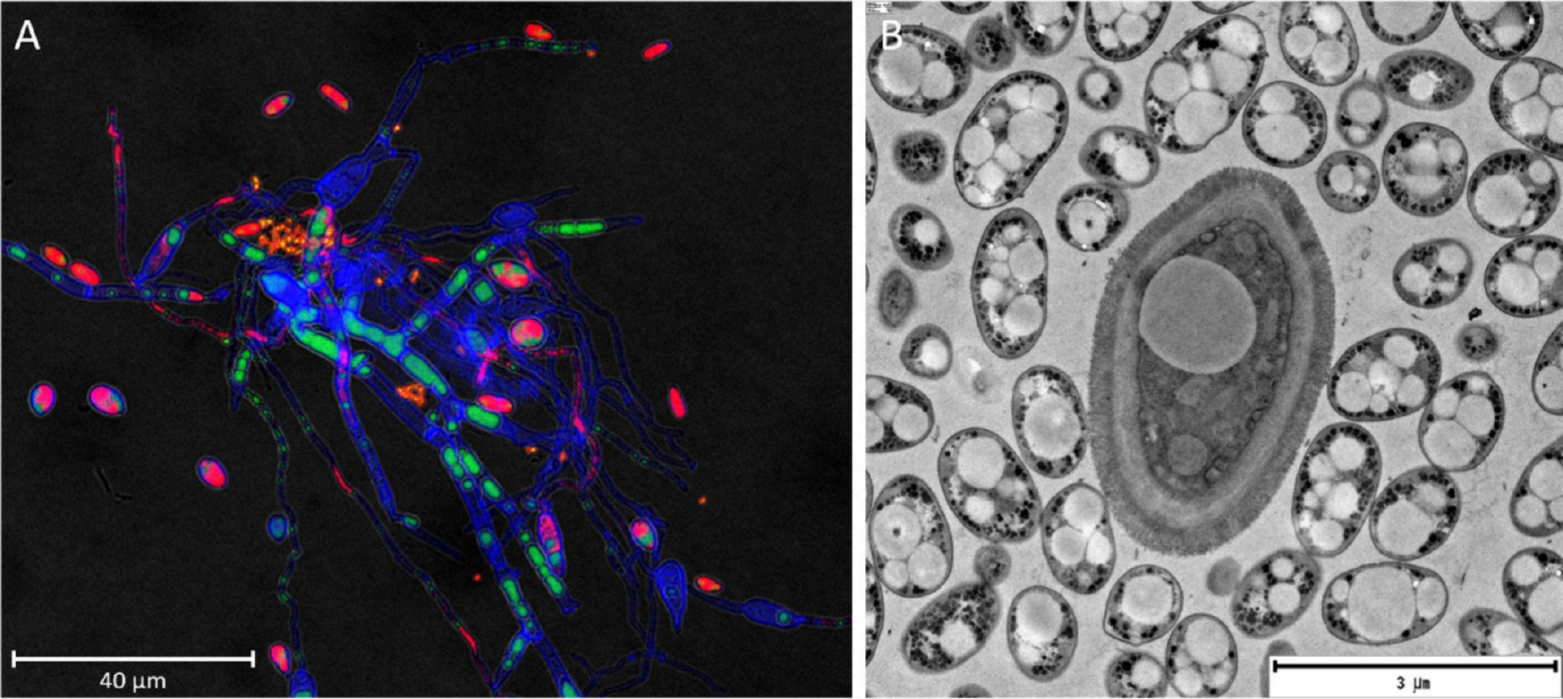
A) CLSM image of the consortium *O. piceae-P. putida* induced with farnesol after 1 week of growth in BSG-WCO medium, stained with different fluorochromes. Propidium iodide (red) stained DNA, calcofluor white (blue) binds to chitin from fungal cells (and to cellulose from BSG), and BODIPY (green) labels hydrophobic particles such as PHA granules in *P. putida* or lipid bodies in *O. piceae*. B) TEM image of the farnesol-induced consortium. An *O. piceae* yeast or hyphae is observed in the center of the image surrounded by *P. putida* cells containing PHA granules in the cytoplasm.

On the other hand, the proportion of planktonic yeast vs bacteria in the cocultures was evaluated by flow cytometry after filtration. The yeast/bacteria ratio decreased notably from initial values of around 70 at 48 h, independently of farnesol induction, to 5.6 ± 1.3 yeast per bacterial cells in the noninduced and 3.2 ± 0.2 in the induced consortia at 192 h. The yeast and bacteria count indicated that farnesol increased the bacteria population in the consortium (4.53·10^5^ cells·mL^–1^ and 7.54·10^5^ cells·mL^–1^ at 192 h in noninduced and induced consortia, respectively).

### Production of PHAs

One of the main components of the substrates used in the fermentations (BSG and WCO) are lipids, which can be utilized by the microorganisms to produce biomass and, in the case of *P. putida,* to accumulate PHAs. In particular, the culture medium was very rich in triglycerides that can be hydrolyzed to release free fatty acids and glycerol by the action of lipases. Thus, the presence of extracellular lipolytic activity was evaluated in each culture using a generic esterase assay at two sampling points (96 and 168 h) (Table 1). No esterase activity was detected in *P. putida* cultures with or without farnesol at any sampling time. However, high activity levels were observed in *O. piceae* as it secretes a versatile lipase (OPE), which is the only extracellular esterase activity in this fungus.^31^ This enzyme is very efficient at hydrolyzing triglycerides and sterol esters,^29^ such as those present in WCO and BSG. Induction with farnesol showed a clear effect on the esterase activity in *Ophiostoma* monocultures, while in the consortium it seems to be already induced by presence of the bacteria and the addition of the QSM did not show such a marked effect (Table 1).

**Table 1:** Esterase activity on *p*NPB (mU·mL^–1^) detected in supernatants of monocultures and cocultures induced or not with farnesol after 96 and 168 h of growth

Culture	96 h	168 h
*O. piceae*	88.9 ± 6.7	112.3 ± 11.5
*O. piceae* + farnesol	126.7 ± 6.4	186.4.0 ± 3.6
Consortium	45.0 ± 0.8	183.1 ± 2.2
Consortium + farnesol	37.8 ± 1.8	190.8 ± 6.5

In this sense, the compounds released from lipid hydrolysis can constitute an excellent substrate for PHAs production by *P. putida*.^49^ The amount of PHA produced was measured at the end of the flask-shake experiments (192 h) (Table 2). In the case of *O. piceae*, no production was detected since it does not have the metabolic pathways involved in the biosynthesis of PHAs. *P. putida* monoculture did not accumulate PHA, since the bacteria is not able to grow efficiently on BSG and WCO. Total PHA production in the cocultures was enhanced by addition of farnesol (1.4 vs 1.9 g·L^–1^), corresponding with a PHA accumulation percentage of 8.0% and 10.4% respectively. Since the percentage of PHAs was referred to the total biomass of the consortium, including the residues, to analyze the actual percentage of accumulation in the bacterium, we filtrated the cultures through a 3 μm filter, which allowed the separation and recovery of most bacteria and the evaluation of their PHA content. *P. putida* was able to accumulate ≈ 40% of PHAs both in the induced and noninduced consortium. These values are comparable to other works in which *P. putida* KT2440 produces PHA from chemically hydrolyzed WCO or crude glycerol with a genetically engineered strain, obtaining 36.4 ^50^ and 38.9%^51^ of PHA referred to whole biomass in the reactor, respectively. These data, together with the results on the population analysis carried out by flow cytometry, indicates that farnesol induction improves *P. putida* growth in the consortium, indirectly increasing PHA production, but do not increase PHA content per cell (Table 2).

**Table 2:** PHA content in cultures and co-cultures of 192 h. The content is presented as C_PHA_: Mass PHA proportion (total biomass basis, g_*x*_), Mass PHA proportion after 3 μm filtration (filtered biomass basis, g_x(3μm)_) and total PHA concentration (reaction volume basis) represented as a percentage

Culture	C_PHA_		
	g_**PHA**_ **·g**_*x*_^**–1**^	g_**PHA**_ **·g**_**x(3um)**_^**–1**^	g_**PHA**_ **·L** ^**–1**^
*P. putida*	0.1 ± 0.0	0.4 ± 0.2	0.0 ± 0.0
*P. putida +* farnesol	0.1 ± 0.0	0.5 ± 0.0	0.0 ± 0.0
*O. piceae*	0	0	0
*O. piceae* + farnesol	0	0	0
Consortium	8.0 ± 1.2	42.4 ± 0.8	1.4 ± 0.3
Consortium + farnesol	10.4 ± 1.5	40.1 ± 3.4	1.9 ± 0.4

Other complex feedstocks have been studied for PHA production. For example, *Pseudomonas* species synthesized 20–30% of PHA in dry weight using pretreated and hydrolyzed perennial ryegrass,^52^ and *Haloferax mediterranei* 2.12 g·L^–1^ of PHA utilizing hydrolysates of macroalgal biomass.^53^ However, there is only one report on the production of PHAs from plant biomass using microbial consortia. Saratale et al.^54^ showed that a bacterial coculture of *Lysinibacillus* sp. RGS and *Ralstonia eutropha* ATCC 17699, grown using enzymatic hydrolysates of acid-pretreated sugar cane bagasse as carbon source, was able to produce up to 11 g·L^–1^ of PHA in 48 h. Nevertheless, pretreatment and saccharification of the feedstock are required in all these cases, which makes the difference with the interkingdom consortium formed by *O. piceae* and *P. putida*. In our case, *O. piceae* provides the enzymes required to degrade the substrates, liberating compounds that *P. putida* can metabolize, which avoids the need for saccharification, a step that dramatically increases costs at industrial scale. Thus, taking advantage of their complementary activities, this consortium has a promising potential for industrial production of PHA from untreated lignocellulosic biomass.

### Transcriptomics and metabolomics analysis

Omics analyses of the monocultures and the consortium, with and without farnesol, provided information about the trophic interactions between the microorganisms and the PHA production. All processed data can be consulted in an interactive Shiny app (https://jgf-bioinformatics.shinyapps.io/Visualization_app/) for an easy and comprehensive analysis of all the data set generated. This application allows users to navigate through all of the transcriptomic and metabolic processed data easily using different filters and search for specific genes and metabolites to explore its changes through the different experimental conditions. The code used for the development of this application (see Materials and Methods) could help other researchers develop similar user-friendly applications that help in data analysis and interpretation.

It has been described that, in *O. piceae*, farnesol induces an improved expression of the genes that code for several hydrolases, including the versatile lipase OPE.^28,31^ This lipase is relevant for deconstruction of the residues used as carbon sources in this study, since it hydrolyzes tri, di and monoacylglycerols and sterol esters.^30^ The increase in enzymatic activity was associated with the induction of filamentation by the QSM, as hyphae are more efficient at secreting enzymes than yeast.^55^ Here, transcriptional analysis of *O. piceae* monocultures showed the differential expression of the OPE genes when farnesol is added (log2 FC > 1.5). On the other hand, analysis of fungal gene expression in consortia with and without farnesol showed higher induction of activities related to BSG degradation, annotated as glycosyl hydrolases, oxidoreductases, proteases, and peptidases (Figure 4). This effect could be attributed to the presence of *P. putida* and the induction of filamentation in the fungus by its QS molecule. Specifically, an increase in the esterase activity (Table 1) and the transcriptomic levels of OPE (log2 FC of 2.4) was observed in noninduced cocultures grown for 168 h.

**Figure 4:**
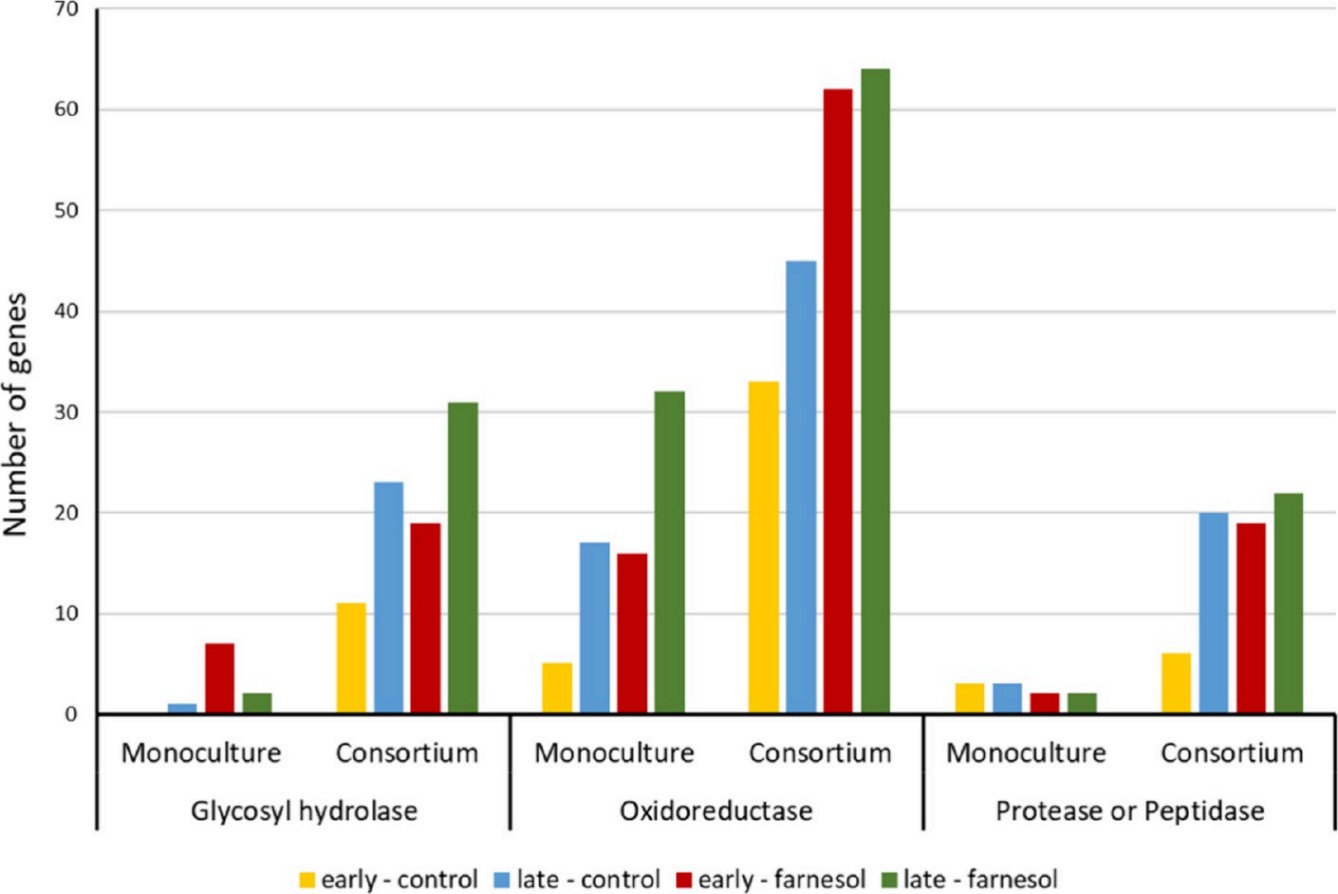
Number of genes annotated in the classes “glycosyl hydrolase”, “oxidoreductase” and “protease or peptidase” identified in the *O. piceae* monoculture and the consortia. Each bar corresponds to a different experimental condition, being the early sampling point (96 h) of noninduced cultures in yellow, the late sampling point (168 h) of noninduced cultures in blue, the early sampling point (96 h) of farnesol induced cultures in in red, and in the late sampling point (168 h) of farnesol induced cultures in green.

Thus, the activation of degrading enzymes will presumably result in higher availability of assimilable monomers for both microorganisms, boosting their growth and metabolic activity. For example, metabolomic analysis of cultures and cocultures of *O. piceae* showed a reduction in the class “glycerolipids” (including acylglycerols) as the OPE breaks them down. However, a concomitant increase in the abundance of free fatty acids and glycerol was not detected, because these molecules are presumably being consumed. Indeed, transcriptomics data revealed the overexpression in *P. putida* of the pathway involved in glycerol metabolism. The glycerol catabolic pathway (formed by the transporter PP_1076, the glycerol kinase PP_1075 and the glycerol-3-phosphate dehydrogenase PP_1073) was more heavily expressed in the consortia with or without farnesol than in *P. putida* monocultures (log2 FC of 3.2, 1.3, and 2.7 respectively), while the repressor *glpR* of the operon is downregulated (log2 FC of −1.4).

A similar situation was observed for citric acid, independent of farnesol induction. D’arrigo et al.^56^ described the enhanced expression of some transporters (the predicted porin PP_1419, the gene cluster for the tricarboxylate transporter TctABC PP_1418–16, and the gene encoding the putative citrate transporter PP_0147) during the growth of *P. putida* on citrate. In accordance with these results, our data showed higher expression of these genes in the consortia compared to the bacterial monoculture since, as other fungi, *O. piceae* produces citric acid during its growth.^57,58^ This is supported by the greater representation of the metabolic subclass “tricarboxylic acids” (which are mainly composed of citric and isocitric acids) in the metabolomes of cultures in which *O. piceae* was present. Therefore, these data suggest that the citrate released by the fungus can be consumed by the bacterium.

Regarding nitrogen metabolism, the analysis of the GO terms of *P. putida* in the consortia showed an enriched expression pattern in categories related to urea (Figure S1 E-H) and amino acid (Figure S1 E) transport and nitrogen metabolism (nitrite reductase) (Figure S1 F-H) compared to the monoculture. Thus, the fungus should be providing the bacteria with an assimilable nitrogen source. Going back to *O. piceae,* the enhanced protease activity detected in the consortium (Figure 4) may release amino acids from the proteins of BSG, producing urea as a metabolic subproduct. Both, amino acids and urea are good nitrogen sources for *P. putida* growth. In this consortium, nitrogen could be playing an important role in microbial interaction, as described in other natural communities.^59–61^ Similarly, the analysis of enriched GO terms in *O. piceae* showed the overexpression of genes related to iron binding (Figure S1 A-D) and ferric-chelate reductase activity (Figure S1. A and D). Iron is an important driver in microbial interactions^62^ and our data suggest its relevance in the sein of our consortium.

Finally, the expression of genes involved in the metabolic pathways that lead to PHA production by *P. putida* KT2440 were also analyzed. The *pha* gene cluster is well conserved among the mcl-PHA producer strains and contains 2 operons. phaC1ZC2D and phaFI^63^ encode for two PHA synthases (*phaC1* and *phaC2*), a PHA depolymerase (*phaZ*), a transcriptional regulator of the cluster (*phaD*) and two phasins (*phaI* and *phaF*).^64^ Transcriptomic analysis disclosed the overexpression of *phaIF* genes (log2 FC of 1.8 and 4.2 respectively) and the repression of the depolymerase *phaZ* (log2 FC of −1) in both consortia compared to the monoculture. These data are consistent with higher PHA production in the consortium (Table 2). When PHAs are synthesized from nonstructurally related substrates, these are metabolized to acetyl-CoA, channelled to the fatty acid biosynthesis (FAB) pathway and transformed into polymerizable PHA monomers by the gene *phaG* (PP_1408).^65^ High expression levels of this gene were observed in both consortia compared to *P. putida* monocultures (log2 FC of 6.4) as well as the whole FAB pathway. These outcomes suggest that PHA production may be mostly derived from nonstructurally related substrates from 96 h onward, indicating that fatty acids liberated by OPE had already been consumed.

In addition, metabolomic analysis showed that the subclass “medium chain hydroxyacids and derivatives”, involving PHA monomers such as 3-hydroxyoctanoic acid, 3-hydroxydecanoic acid, and 3-hydroxydodecanoic acid, was very abundant in the consortia (peak height 10^9.1^) compared to both monocultures (10^7.5^ and 10^8.5^ for *P. putida* and *O. piceae*, respectively). In Pseudomonads, PHA-structurally related substrates like these ones are rapidly polymerized into PHA through intermediates of the β-oxidation pathway,^49^ which could explain higher PHA production in the consortium.

### Scale-up to bioreactor

The above results demonstrate the feasibility of producing PHAs from waste at the flask scale through the consolidated bioprocess designed in this work using the *P. putida – O. piceae* consortium. As a proof of concept of its scalability, the whole experiment was reproduced in a 5-L bioreactor, programming the same conditions set up for the flask experiments with slight adjustments to maximize PHA production. In this occasion, substrate load (BSG-WCO) was increased to 2% with no sterilization or pretreatment of the wastes, the microbial load was increased to 10^7^ of each microorganism, and both of them were inoculated at the same time, to resemble industrial processing conditions. The bioreactor reached higher optical density and a PHA content of the 13% of total biomass. The PHA concentration increased to 6.7 g·L^–1^ at 86 h of culture, with a volumetric productivity of 0.08 g PHA L^–1^ h^–1^ (Table 3).

**Table 3:** Comparation in performance of PHA production between flask experiments and 86-h cultures of the consortium with 2% BSG-WCO in a bioreactor of 5 L (working volume of 2L). Bacterial growth (OD_600 nm_), Y_PHA_: Mass PHA yield (referred to initial BSG and WCO content), C_PHA_: Mass PHA proportion (total biomass basis, g_*x*_) and total PHA concentration (reaction volume basis), P_PHA_: Mass total productivity

	Time (h)	OD_600 nm_	Y_PHA_	C_PHA_		P_PHA_
			g_PHA_·g_biomass_^–1^	g_PHA_·g_*x*_^–1^	g_PHA_·L^–1^	g_PHA_·L^–1^·h^–1^
Flask	192	37.5	0.10	0.10	1.9	0.01
Bioreactor	86	63.5	0.17	0.13	6.7	0.08
% increase			70	25	250	700

These values represent a huge improvement compared to the production of PHA in Erlenmeyer flasks. The PHA yield, calculated based on the total biomass recovered from the culture at 86 h, increased by 70%, and the final Bioplastic concentration rose 250% (Table 3). This enhancement can be attributed to the higher amount of microbial biomass obtained under these conditions, probably due to the increase in both the substrate load and inoculum density. The mass PHA yield, relative to the total feedstock biomass (BSG and WCO) added to the reactor, reached 0.17 g_PHA_·g_WCO_^–1^. It is important to note that the substrate that is probably being transformed into PHA more efficiently is WCO, while BSG may act as a support matrix for the catalytic consortium. When considering only the WCO biomass added (2% v/v, density 0.92 g·mL^–1^) this yield increases to 0.36 g_PHA_·g_WCO_^–1^.

The final PHA concentration and volumetric productivity reach 6.7 g·L^–1^ and 0.08 g_PHA_·L^–1^·h^–1^, respectively. Although these results are lower than those reported in studies employing pretreated or hydrolyzed lipid-derived substrates,^50,51^ they surpass those obtained using untreated lipidic feedstocks^66^ with *P. putida* KT2440 monocultures. Since lignocellulosic substrates require pretreatment prior fermentation with *P. putida*, several studies have addressed this issue,^67,68^ however PHA concentrations achieved with pretreated (alkali, liquid hot water, or diluted acid pretreatment) and/or saccharified biomass are lower those obtained in this study.

Technoeconomical analysis of industrial bioprocesses is necessary for assessing its viability. The bioprocess here presented does not require substrate sterilization, pretreatment or hydrolysis for PHA synthesis, BSG and WCO. In chemical refineries for biodiesel and epoxy fatty acid methyl ester production, WCO hydrolysis via lipase can account up to 7% and 21% of total energy and equipment cost, respectively.^69^ In the case of lignocellulosic biomass biorefinery, usually milling, drilling, and enzymatic hydrolysis are required steps for bioproduction. In the bioprocess presented in this work none of these steps are required, in this way, eliminating these steps in a hypothetical biorefinery producing bioethanol, 2,3-butanediol and PHA from BSG^70^ could result in saving approximately 50% of total equipment expenses. However, other feedstocks such as wheat straw may need milling and drilling in order to increase its surface for the catalytic biofilm formation. Additionally, energetic cost of the process are expected to be lower as enzymatic saccharification is highly inefficient, leading to energetic loses up to 52%.^70^

To sum up, the results from this work are very promising since high PHA production values were achieved in shorter times than at flask scale and in conditions close to those used in an industrial environment, using secondary raw materials that were not pretreated or sterilized. Thus, the combination of increasing PHA production while reducing industrial costs paves the way for the optimization of this consolidated bioprocess and its industrial scale-up.

## Conclusions

This work demonstrates that the establishment of an interkingdom microbial consortium between the fungus *O. piceae* and the bacterium *P. putida* allows the valorization of two complex residues (BSG and WCO) in a single biotechnological process, without the need of pretreatment or enzymatic hydrolysis. In this setup, the fungus secretes the enzymes necessary to release monomers assimilable by the bacteria from the secondary raw materials provided as nutrients, which *P. putida* uses to grow and produce PHA. This consolidated bioprocess approach constitutes an unbeatable alternative for the bioconversion of complex waste into value-added products in the current framework of the circular economy.

The multiomics analysis of the consortium allowed a deeper understanding of the microbial interactions involved in the process, bringing up potential optimization targets and metabolic improvements for increased PHA production. The interactive Shiny app created for the interpretation of the dual RNA-seq and metabolomic results provides a user-friendly platform for researchers to explore and analyze the complex data set generated in these experiments, improving the accessibility and interpretability of these findings.

Finally, the scaling up of the bioprocess to a 5 L bioreactor has served as a proof of concept for the optimization of PHA production and as the first step toward the industrial implementation of this consolidated bioprocess.

## Supplementary Material







## Notes

Overexpressed fungal and bacterial GO terms in consortium vs monocultures under farnesol-induced and noninduced conditions (PDF)
