# Supplementary figures and images for: Quorum-driven microbial consortium for Bioplastic production from agro-waste
Source: ACS Sustain Chem Eng. 2025 Aug 28;13(36):15038–49. doi: 10.1021/acssuschemeng.5c05453 (PMC12442501; doi:10.1021/acssuschemeng.5c05453)

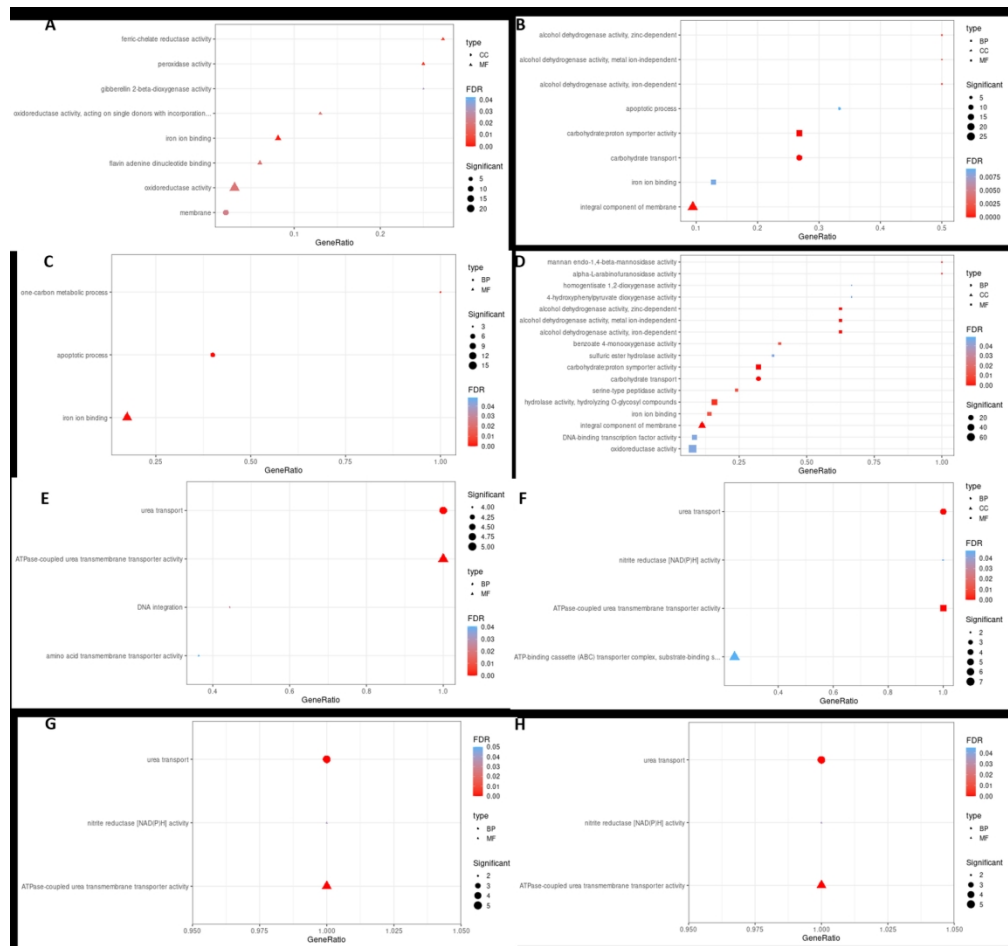

Supplement: Supplementary file 1 [file sc5c05453_si_001.pdf]
