# Supplementary material for: Quorum-driven microbial consortium for Bioplastic production from agro-waste
Source: ACS Sustain Chem Eng. 2025 Aug 28;13(36):15038–49. doi: 10.1021/acssuschemeng.5c05453 (PMC12442501; doi:10.1021/acssuschemeng.5c05453)
Supplement: Supplementary file 2 [file sc5c05453_si_002.pdf]

Supporting information for

Quorum-driven microbial consortium for bioplastic production from agro-waste

Diego Crespo-Roche[1], Marta Herráez[1], Javier Guerrero-Flores[1], M. Jesús Martínez[1], Katherine Louie[2], Trent Northen[2], Alicia Prieto[1], Jorge Barriuso[1]*

[1]Centro de Investigaciones Biológicas Margarita Salas, Consejo Superior de Investigaciones Científicas (CIB-CSIC). C/ Ramiro de Maeztu 9, 28040 Madrid, Spain.
[2]Joint Genome Institute, Lawrence Berkeley National Laboratory, Berkeley, CA 94720, USA

*Corresponding author: Jorge Barriuso. Centro de Investigaciones Biológicas (CIB Margarita Salas-CSIC), Ramiro de Maeztu 9, E-28040 Madrid, Spain. Tel.: +34 918373112; fax: +34 915360432. E-mail address: jbarriuso@cib.csic.es

Number of pages: 3

Number of figures: 1

Number of tables: 0

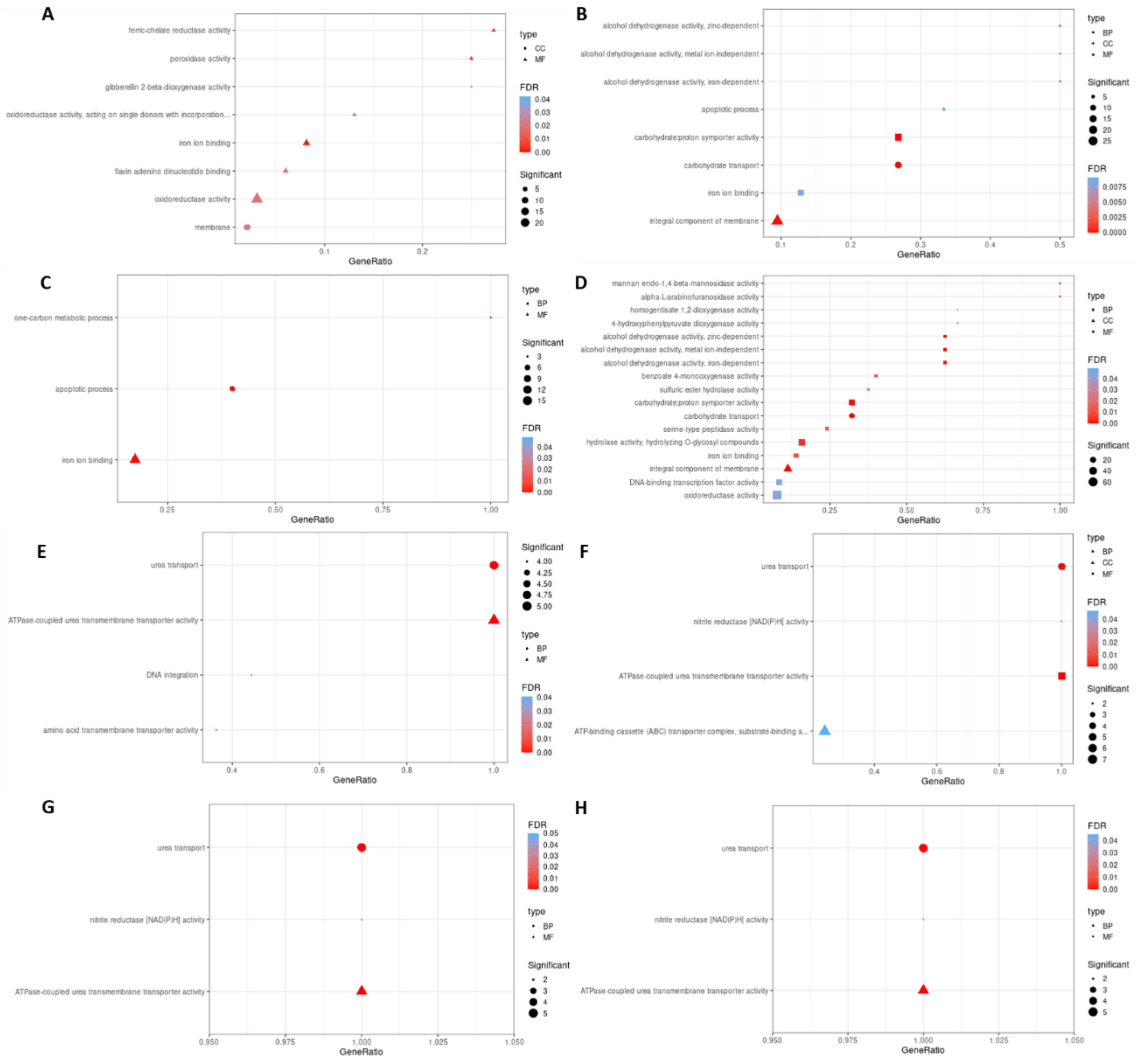

Figure S1. Fungal and bacterial GO terms overexpressed in the consortium compared to the corresponding monoculture. The samples analyzed were taken at two sampling times from cultures induced or not by farnesol. (A-D) *O. piceae* genes: A) non-induced, 96 h; (B) non-induced, 168 h; (C) induced, 96 h; (D) induced, 168 h. (E-H) *P. putida* genes: (E) non-induced, 96 h; (F) non-induced, 168 h; (G) induced, 96 h; (H) induced, 168 h. Each point corresponds to a differentially expressed GO term (● biological process, ■ cellular component, ▲ molecular function), each color corresponds to a *p*-value (from 0.05 in blue to 0.00 in red), the point size is proportional to the number of genes differentially expressed in each GO term, and the gene ratio is the quotient between the

differentially expressed genes in a GO term in a given condition and all the genes in this GO term found in the same condition.
