# Supplementary material for: Quorum-driven microbial consortium for Bioplastic production from agro-waste
Source: ACS Sustain Chem Eng. 2025 Aug 28;13(36):15038–49. doi: 10.1021/acssuschemeng.5c05453 (PMC12442501; doi:10.1021/acssuschemeng.5c05453)
Supplement: Supplementary file 3 [file sc5c05453_si_003.pdf]

Supplementary material

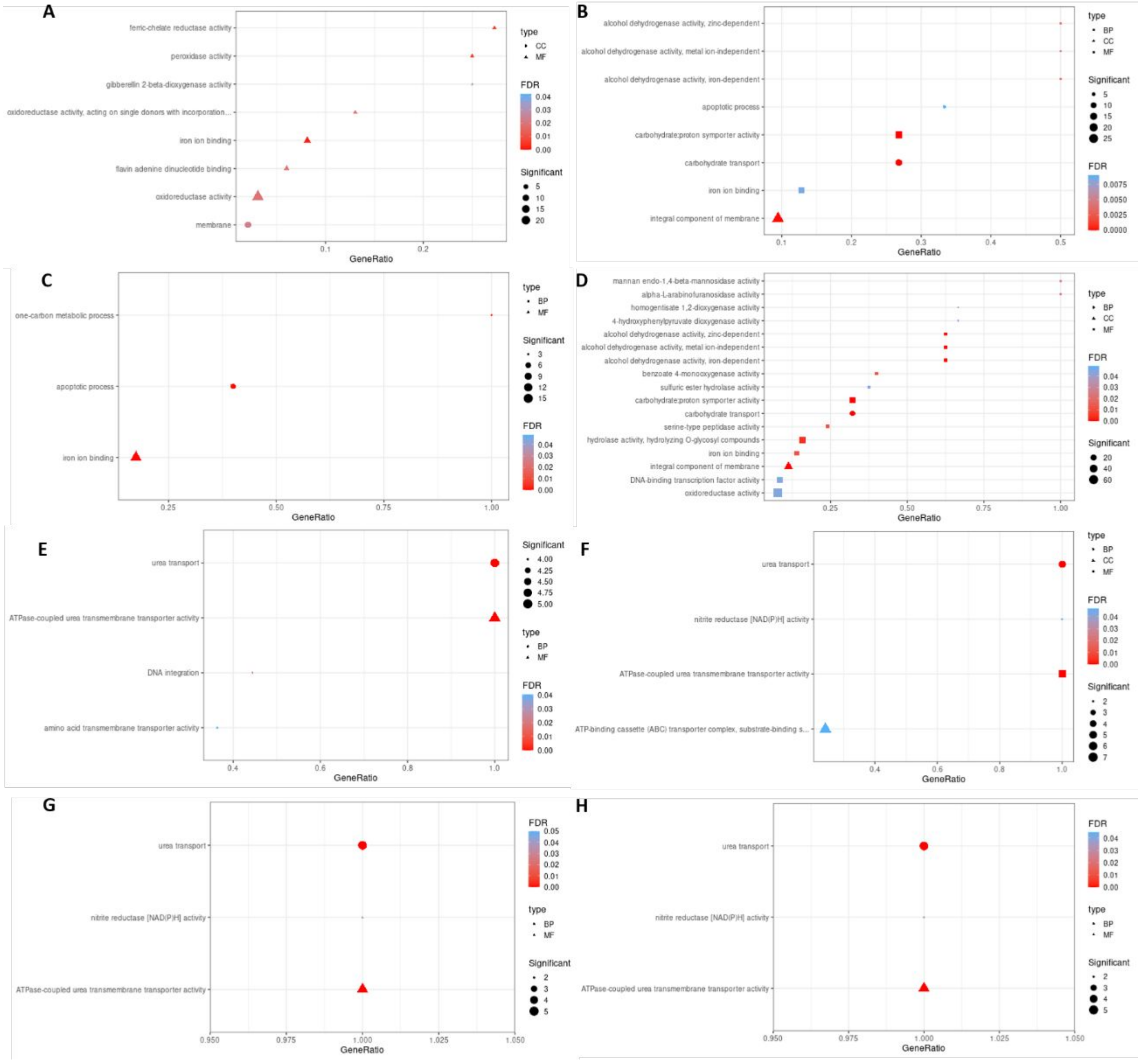

Figure S1. Fungal and bacterial GO terms overexpressed in the consortium compared to the corresponding monoculture. The samples analyzed were taken at two sampling times from cultures induced or not by farnesol. (A-D) *O. piceae* genes: A) non-induced, 96 h; (B) non-induced, 168 h; (C) induced, 96 h; (D) induced, 168 h. (E-H) *P. putida* genes: (E) non-induced, 96 h; (F) non-induced, 168 h; (G) induced, 96 h; (H) induced, 168 h. Each point corresponds to a differentially expressed GO term (● biological process, ■ cellular component, ▲ molecular function), each color corresponds to a *p*-value (from 0.05 in blue to 0.00 in red), the point size is proportional to the number of genes differentially expressed in each GO term, and the gene ratio is the quotient between the

differentially expressed genes in a GO term in a given condition and all the genes in this GO term found in the same condition.
